# Glycated Haemoglobin Is Associated With Poorer Cognitive Performance in Patients With Recent-Onset Psychosis

**DOI:** 10.3389/fpsyt.2020.00455

**Published:** 2020-05-25

**Authors:** Itziar Montalvo, Alexandre González-Rodríguez, Ángel Cabezas, Alfonso Gutiérrez-Zotes, Montse Solé, Maria José Algora, Laura Ortega, Lourdes Martorell, Vanessa Sánchez-Gistau, Elisabet Vilella, Javier Labad

**Affiliations:** ^1^Department of Mental Health, Parc Taulí Hospital Universitari, Institut d’Investigació Sanitària Parc Taulí (I3PT), Universitat Autònoma de Barcelona, CIBERSAM, Sabadell, Spain; ^2^Hospital Universitari Institut Pere Mata, Institut d’Investigació Sanitària Pere Virgili (IISPV), Universitat Rovira i Virgili, CIBERSAM, Reus, Spain; ^3^Nursing Department, Universitat Rovira i Vigili, Tarragona, Spain

**Keywords:** glucose, glycated haemoglobin, cognition, early psychosis, cortisol

## Abstract

**Background:**

Glucose abnormalities and cognitive alterations are present before the onset of schizophrenia. We aimed to study whether glucose metabolism parameters are associated with cognitive functioning in recent-onset psychosis (ROP) patients while adjusting for hypothalamic-pituitary adrenal (HPA) axis measures.

**Methods:**

Sixty ROP outpatients and 50 healthy subjects (HS) were studied. Cognitive function was assessed with the MATRICS Consensus Cognitive Battery. Glycated haemoglobin (HbA1_c_), glucose, insulin, and C-peptide levels were determined in plasma. The HOMA-insulin resistance index was calculated. Salivary samples were obtained at home on another day to assess the cortisol awakening response and cortisol levels during the day. Univariate analyses were conducted to explore the association between glucose metabolism parameters and cognitive tasks. For those parameters that were more clearly associated with the cognitive outcome, multiple linear regression analyses were conducted to adjust for covariates. Each cognitive task was considered the dependent variable. Covariates were age, sex, education level, diagnosis, antipsychotic and benzodiazepine treatment, body mass index (BMI), smoking, and HPA axis measures. Potential interactions between diagnosis and glucose parameters were tested.

**Results:**

There were no significant differences in HPA axis measures or glucose parameters, with the exception of C-peptide (that was higher in ROP patients), between groups. ROP patients had a lower performance than HS in all cognitive tasks (p < 0.01 for all tasks). Of all glucose metabolism parameters, HbA1c levels were more clearly associated with cognitive impairment in cognitive tasks dealing with executive functions and visual memory in both ROP patients and HS. Multivariate analyses found a significant negative association between HbA1c and cognitive functioning in five cognitive tasks dealing with executive functions, visual memory and attention/vigilance (a ROP diagnosis by HbA1_c_ negative interaction was found in this latter cognitive domain, suggesting that HBA1_c_ levels are associated with impaired attention only in ROP patients).

**Conclusions:**

Our study found that HbA1_c_ was negatively associated with cognitive functioning in both ROP patients and HS in tasks dealing with executive functions and visual memory. In ROP patients, HbA1_c_ was also associated with impaired attention. These results were independent of BMI and measures of HPA axis activity.

## Introduction

Cognitive alterations are well-known predictors of social functioning in people with schizophrenia and related psychotic disorders (1). These cognitive alterations are present at early stages of the psychotic illness, even before the development of positive psychotic symptoms (delusions, hallucinations) (2). Biological mechanisms explaining these cognitive deficits are complex and include the potential role of hypothalamic-pituitary-adrenal (HPA) axis hormones (3, 4), thyroid hormones (5, 6), prolactin (7, 8), inflammatory markers (9, 10) and the genetic background (11).

In our current study, we aimed to explore whether glucose metabolism parameters might contribute to the cognitive impairment of people with recent-onset psychosis (ROP). Previous studies including drug-naïve first-episode psychosis and healthy controls have reported increased glucose and insulin resistance (12) and impaired glucose tolerance (13), suggesting that glucose-related parameters may be altered in patients with psychosis at the early stages of the illness. Previous studies have also reported an increased prevalence of type 2 diabetes in the parents of people with non-affective psychosis (14). It was initially thought that this association may be due to shared environmental or genetic risk factors, or both. However, a recent study (15) exploring the association between polygenic risk score of schizophrenia and glycated haemoglobin (HbA1_c_) while adjusting for polygenic risk score of type 2 diabetes, and clinical and demographic covariables suggests that the mechanisms of hyperglycemia or diabetes are at least partly independent from genetic predisposition to schizophrenia.

It is also known that comorbidity with diabetes mellitus is associated with more severe cognitive deficits in schizophrenia (16). Type 2 diabetes is a risk factor for cognitive decline (17), mild cognitive impairment (18), and progression to dementia (19). Although the exact pathophysiology of mild cognitive impairment in type 2 diabetes is unclear, many studies suggest that several coexisting risk factors contribute to the cognitive impairment (20): chronic hyperglycaemia, diabetic complications (macrovascular and microvascular disease), inflammatory reaction and advanced glycation end products, and psychological status (e.g. depressive symptoms). In a systematic review (21) that included 86 studies exploring the role of glucose regulation (glycaemia, hypoglycaemic events, insulin concentration, insulin resistance, and glucose-lowering treatment) and cognitive function in people with type 2 diabetes without dementia, high HbA1_c_ and glucose variability were negatively associated with cognitive function. HbA1_c_ is a promising biomarker for cognitive impairment because it is also associated with poorer cognitive abilities in people without diabetes (17, 22). A recent study suggests that HbA1_c_ is associated to both cognitive performance and white matter integrity in healthy young adults (22).

Few studies have explored the potential relationship between glucose metabolism indices and cognitive functioning in patients with psychotic disorders. In a study conducted in first-episode drug-naïve patients with schizophrenia, glucose intolerance (measured with a 75 g oral glucose tolerance test) was associated with more negative symptoms and poorer social cognition, but not with poorer neurocognitive performance (23). In another study that measured HbA1_c_, this parameter was associated with poorer global cognition and attention in men (but not women) with schizophrenia (24). Insulin resistance has been associated with alterations in dopaminergic reward systems and homeostatic signals affecting food intake, glucose metabolism, body weight, and cognitive performance (25), being a potential moderator of the cognitive outcome in patients with psychotic disorders. Although several studies have reported an association between diabetes and cognition on schizophrenia, few investigations have explored the role of glucose parameters on cognition in patients with early psychosis.

We aimed to explore the previous hypothesis while adjusting for HPA axis measures, as some indices, such as a blunted cortisol awakening response (CAR) (3, 26, 27) and elevated cortisol diurnal levels during the day (26), have been reported to be associated with a poorer cognitive outcome in people with ROP. Moreover, elevated glucocorticoids contribute to the cognitive impairment of patients with type 2 diabetes (28).

Taking into account that HbA1_c_ has been associated with poorer cognitive performance in both patients with schizophrenia (24) and healthy individuals (22), we hypothesized that HbA1_c_ would be associated with poorer cognitive performance in both people with ROP and healthy individuals. We also aimed to conduct exploratory analyses regarding the contribution of other glucose-related parameters (fasting glucose, c-peptide, insulin resistance) on poorer cognitive functioning.

## Methods

### Sample

Sixty ROP outpatients and 50 healthy subjects (HS) were studied. All patients (aged between 18 and 35 years) were attending the Early Intervention Service for Psychosis from Reus (Hospital Universitari Institut Pere Mata, Spain) had a DSM-IV diagnosis of a psychotic disorder [schizophreniform disorder (n=14); schizophrenia (n=10); schizoaffective disorder (n=8) or a psychotic disorder not otherwise specified (n=28)]. All patients had a duration of illness of <3 years (65% were patients with first-episode psychosis). A control population of 50 HS matched by sex and age was recruited from the community using advertisements. The sample of the study belongs to a project aiming to study the relationship between hormones and cognitive abilities in early psychosis. For this reason, participants of our study participated in a previous study that tested a different hypothesis focused on HPA axis hormones (3). The exclusion criteria were severe neurological disease or mental retardation; pregnancy; language difficulties; visual impairment; alcohol, heroin or cocaine dependence; or treatment with glucocorticoids.

The research protocol was approved by the Ethics Committee of Hospital Universitari Sant Joan, and all participants provided written informed consent after having received a full explanation of the study.

### Clinical Assessment

All patients were interviewed by an experienced psychiatrist using the Schedules for Clinical Assessment in Neuropsychiatry (29). The OPCRIT checklist version 4.0 (available at http://sgdp.iop.kcl.ac.uk/opcrit/) was used to obtain DSM-IV diagnoses. The severity of positive, negative and general symptoms was assessed with the Positive and Negative Syndrome Scale (PANSS) (30, 31) to assess the severity of psychotic symptoms.

The Spanish version of the MATRICS Consensus Cognitive Battery (MCCB) was used to assess neurocognitive functioning (32), and it includes 10 cognitive tests assessing 7 cognitive domains: processing speed, attention and vigilance, working memory, verbal learning, visual learning, reasoning and problem solving, and social cognition.

Sociodemographic and clinical variables were obtained in a semi-structured interview. Substance use was recorded as the consumption of alcohol (standard units/day), tobacco (cigarettes/day), and cannabis (joints/day). Current psychopharmacological treatment was recorded during the neuropsychological assessment, as described previously (3). The dose of antipsychotics was converted to chlorpromazine equivalents following an international consensus of antipsychotic dosing (33).

Weight, height, waist circumference, and blood pressure were assessed by physical examination. Body mass index (BMI) was calculated with the formula weight (kg)/height (m)^2^.

### Biochemical Measures

A fasting morning blood analysis (between 8:30 a.m. and 9:30 a.m.) was obtained by antecubital venepuncture. HbA1_c_, glucose, insulin, and C-peptide were determined in plasma. The Homeostatic Model Assessment for Insulin Resistance (HOMA-IR) index was calculated using the formula HOMA-IR = [insulin (µUI/mL) x glucose (mg/dL)]/405.

Salivary samples were obtained at home on another day with Salivette^®^ tubes to assess the cortisol awakening response (calculated as the area under the curve with respect to the increase, considering three samples: awakening, 30’ post-awakening, and 60’ post-awakening) and cortisol levels during the day (calculated as the area under the curve with respect to the ground, considering 5 samples: awakening, 30’ post-awakening, 60’ post-awakening, 10:00 a.m., and 11:00 p.m.). Both formulas were computed using the trapezoid formula (34). A full explanation of the processing of the samples and cortisol determination with a high-sensitivity enzyme-linked immunosorbent assay (ELISA) kit has been described elsewhere (3).

### Statistical Analyses

We used SPSS v 23.0 for conducting statistical analyses. Cortisol measures were transformed with a Box-Cox transformation (35), and the Trail Making Test (part A) was log transformed (ln) to reduce skewness. Chi-squared tests and T-tests were used to compare categorical and continuous data between both diagnostic groups. Non-parametric tests (Mann Whitney U test) were used to compare ordinal variables or continuous measures that were skewed (e.g. insulin, c-peptide). Pearson correlation analyses (and Spearman when needed) were used to explore associations between continuous variables. A p value <0.05 (two-tailed) was considered to be significant.

As HbA1_c_ was considered the glucose-related parameter to be studied in our main hypothesis, we verified that this value followed a normal distribution and checked for potential outliers with the 1.5 quartile (Q) rule for outliers (36). With this definition, any observation is a suspected outlier if it falls more than 1.5 x interquartile range (IQR) above the third quartile or below the first quartile. In our sample, the distribution for HbA1_c_ values was: minimum value= 4.6, Q1 = 4.9, Q2 (median)= 5.1, Q3 (5.3), maximum value 5.8, IQR= 0.4. Therefore, none of the values were considered outliers because they were within the limits 1.5xIQR (lower interval: 4.3, upper interval: 5.9).

Although our main hypothesis was conducted with multiple linear regression analyses, we first conducted an exploratory and univariate analysis to test the associations between different glucose-related parameters and cognitive outcomes. We also included exploratory correlational analyses between cognitive scores. These exploratory analyses were not adjusted for multiple comparisons following some recommendations that indicate that is it not strictly necessary to correct for multiple testing in analyses that are exploratory in nature (37). Multiple linear regression analyses were conducted for testing the association between HbA1_c_ and cognitive variable while adjusting for covariates. Several multiple linear regression analyses were conducted, considering each cognitive task as the dependent variable. HbA1_c_ was considered the main independent variable. We avoided the inclusion of different glucose metabolism parameters in the same equation because they were highly correlated. The following covariates were included in each equation with the enter procedure: age, sex, education level, diagnosis, antipsychotic and benzodiazepine treatment, BMI, smoking, and HPA axis measures. Potential interactions between diagnosis and HbA1_c_ were tested, and those significant interactions were included in the final model.

### Sample Size and Power Analysis

G* Power 3.1.9.4. was used for sample size and power calculations. The original sample was calculated for detecting a moderate effect size (f^2^ = 0.2) with multiple linear regression analyses, considering an alpha error of 0.05 and a beta error of 0.15 (statistical power of 85%), and 12 predictors. The needed sample size was 108. It is important to note that our sample is small for detecting small effects. Moreover, in the stratified analysis by diagnosis (e.g. correlation analyses), the statistical power can decrease: the statistical power for detecting moderate effect sizes (r= 0.3) was 59% for healthy individuals and 67% for ROP patients.

## Results

Clinical and hormonal variables from the sample are described in **Table 1**. Both groups were well matched in age and sex, although ROP patients had a lower education status and reported more smoking and alcohol consumption. In relation to glucose-related parameters, C-peptide concentrations were higher in ROP patients than in HS. There were no significant differences in HPA axis measures between groups.

**Table 1:** Clinical characteristics and hormonal measures from the sample.

	HS N= 50		ROP patients N=60		p value
Female sex, N (%)	22	44%	21	35%	0.335
Age (years)	23.8	4.8	24.5	5.4	0.465
Education level (years of study)	13.4	2.7	11.3	2.8	**<0.001**
Smoking, N (%)	10	20%	41	68.3%	**<0.001**
Smoking (cig/day), all participants	1.6	4.4	9.0	9.6	**<0.001**
Smoking (cig/day), only smokers	8.0	7.0	13.1	9.0	0.101
Cannabis use, N (%)					
No	38	76%	43	71.7%	0.215
Sporadic	10	20%	9	15%	
Continuous	2	4%	8	13.3%	
Alcohol consumption, N (%)					
No	5	10%	27	45%	**<0.001**
Sporadic	44	88%	28	46.7%	
Continuous	1	2%	5	8.3%	
BMI (kg/m^2^)	22.5	3.2	24.1	4.1	0.053
Antipsychotic treatment: Chlorpromazine equivalents (mg/day)	0.0	0.0	371.1	334.0	**<0.001**
Benzodiazepine treatment: Diazepam equivalents (mg/day)	0.0	0.0	2.6	7.7	**0.021**
**Glucose metabolism parameters**					
Glucose (mg/dL)	78.6	11.2	78.7	11.1	0.956
Insulin (μIU/mL)	6.6	0 to 22.9	9.5	0 to 114	0.595
C-peptide (μg/L)	1.3	0.6 to 4.2	1.6	0.7 to 12.7	**0.003**
HbA1_c_ (%)	5.1	0.3	5.1	0.3	0.250
HOMA-IR	1.4	0 to 4.9	1.3	0 to 26.18	0.802
**HPA axis measures**					
Cortisol at awakening (nmol/L)	14.7	9.0	13.0	8.6	0.381
Cortisol 30’ post-awakening (nmol/L)	23.8	13.4	20.3	10.7	0.342
Cortisol 60’ post-awakening (nmol/L)	21.0	12.8	16.3	7.5	0.234
10:00 a.m.	12.5	7.4	12.0	6.4	0.971
11:00 p.m.	2.7	2.1	3.0	3.0	0.971
CAR (AUC_i_)	39.7	63.6	40.5	53.7	0.942
Cortisol during the day (AUC_g_)	2049.6	837.3	2082.3	733.4	0.834

Data are mean (SD), median (range) or N (%).

Cortisol raw data are shown. However, p values were obtained using transformed cortisol values (Box-Cox transformation).

BMI, Body mass index; HbA1_c_, Glycated haemoglobin; HOMA-IR, Homeostatic Model Assessment for Insulin Resistance; HPA, Hypothalamic-pituitary-adrenal; CAR, Cortisol awakening response; AUC_i_, Area under the curve (calculated with respect to the increase); AUC_g_, Area under the curve (calculated with respect to the ground).

Significant associations (p < 0.05) are represented in bold.

ROP patients had a lower performance in all cognitive tasks than HS (**Table 2**). The correlation matrix between cognitive measures in all participants is described in **Table 3**.

**Table 2:** Cognitive functioning by diagnostic group.

	HS N=50		ROP patients N=60		p value
	Mean	SD	Mean	SD	
Verbal learning and memory					
HVLT-R	27.5	3.8	22.8	4.9	**<0.001**
Visual learning and memory					
BVMT-R	27.0	5.9	19.6	7.2	**<0.001**
Working memory					
WMS-III-SS (nonverbal)	16.2	2.9	14.2	3.7	**0.002**
LNS (verbal)	14.2	2.9	12.0	2.3	**<0.001**
Processing speed					
TMT-A^†^ (seconds)	24.7	8.5	38.2	13.4	**<0.001**
BACS SC	61.9	9.6	45.9	12.3	**<0.001**
Category Fluency	24.1	5.1	18.3	5.2	**<0.001**
Reasoning and problem solving					
NAB Mazes	27.0	5.9	19.6	7.2	**<0.001**
Attention/vigilance					
CPT-IP	2.7	0.6	2.0	0.7	**<0.001**
Social cognition					
MSCEIT-ME	94.4	9.2	86.8	9.8	**<0.001**

^†^As this cognitive test is measured in seconds, higher scores reflect poorer cognitive performance.

HVLT-R, Hopkins Verbal Learning Test-Revised; BVMT-R, Brief Visuospatial Memory Test-Revised; WMS-III-SS, Corsi Block-Tapping Test; Weschler Memory Scale (3rd edition) spatial span; LNS, Letter Number Span; TMT-A, Trail Making Test part A; BACS-SC, Brief Assessment of Cognition in Schizophrenia-Symbol Coding; NAB, Neuropsychological Assessment Battery; CPT-IP, Continuous Performance Test – Identical Pairs; MSCEIT-ME, Mayer-Salovey-Caruso Emotional Intelligence Test – Managing emotions.

Significant associations (p < 0.05) are represented in bold.

**Table 3:** Pearson's correlations studying the relationship between cognitive tasks in 110 participants.

		HVLT-R	BVMT-R	WMS-III SS	LNS	TMT-A^†^	BACS SC	Fluency	NAB Mazes	CPT-IP	MSCEIT-ME
HVLT-R	r	1	0.596	0.469	0.539	-0.601	0.675	0.594	0.256	0.504	0.401
	p value		< 0.001	< 0.001	< 0.001	< 0.001	< 0.001	< 0.001	.007	< 0.001	< 0.001
BVMT-R	r	0.596	1	0.599	0.554	-0.622	0.690	0.624	0.549	0.491	0.168
	p value	< 0.001		< 0.001	< 0.001	< 0.001	< 0.001	< 0.001	< 0.001	< 0.001	0.086
WMS-III SS	r	0.469	0.599	1	0.387	-0.546	0.504	0.417	0.553	0.376	0.300
	p value	< 0.001	< 0.001		< 0.001	< 0.001	< 0.001	< 0.001	< 0.001	< 0.001	0.002
LNS	r	0.539	0.554	0.387	1	-0.536	0.605	0.473	0.367	0.610	0.286
	p value	< 0.001	< 0.001	< 0.001		< 0.001	< 0.001	< 0.001	< 0.001	< 0.001	0.004
TMT-A^†^	r	-0.601	-0.622	-0.546	-0.536	1	-0.707	-0.559	-0.545	-0.573	-0.308
	p value	< 0.001	< 0.001	< 0.001	< 0.001		< 0.001	< 0.001	< 0.001	< 0.001	.001
BACS SC	r	0.675	0.690	0.504	0.605	-0.707	1	0.653	0.494	0.635	0.312
	p value	< 0.001	< 0.001	< 0.001	< 0.001	< 0.001		< 0.001	< 0.001	< 0.001	0.001
Fluency	r	0.594	0.624	0.417	0.473	-0.559	0.653	1	0.495	0.519	0.220
	p value	< 0.001	< 0.001	< 0.001	< 0.001	< 0.001	< 0.001		< 0.001	< 0.001	0.024
NAB Mazes	r	0.256	0.549	0.553	0.367	-0.545	0.494	0.495	1	0.447	0.173
	p value	0.007	< 0.001	< 0.001	< 0.001	< 0.001	< 0.001	< 0.001		< 0.001	0.078
CPT-IP	r	0.504	0.491	0.376	0.610	-0.573	0.635	0.519	0.447	1	0.294
	value	< 0.001	< 0.001	< 0.001	< 0.001	< 0.001	< 0.001	< 0.001	< 0.001		0.002
MSCEIT-ME	r	0.401	0.168	0.300	0.286	-0.308	0.312	0.220	0.173	0.294	1
	p value	< 0.001	0.086	0.002	0.004	0.001	0.001	0.024	0.078	0.002	

^†^As TMT-A is measured in seconds, greater scores in this variable reflect poorer cognitive performance. For this reason, this variable showed negative correlations with other cognitive tasks. The log transformed variable (ln TMT-A) was used.

HVLT-R, Hopkins Verbal Learning Test-Revised; BVMT-R, Brief Visuospatial Memory Test-Revised; WMS-III-SS, Corsi Block-Tapping Test; Weschler Memory Scale (3rd edition) spatial span; LNS, Letter Number Span; TMT-A, Trail Making Test part A; BACS-SC, Brief Assessment of Cognition in Schizophrenia-Symbol Coding; NAB, Neuropsychological Assessment Battery; CPT-IP, Continuous Performance Test – Identical Pairs; MSCEIT-ME, Mayer-Salovey-Caruso Emotional Intelligence Test – Managing emotions.

Of all glucose metabolism parameters, HbA1_c_ levels were more clearly associated with cognitive impairment in cognitive tasks dealing with executive functions and visual memory in both ROP patients and HS (**Table 4**).

**Table 4:** Correlations between glucose-related parameters and cognitive tasks: Stratified analyses by diagnosis.

		HS					ROP patients				
		HbA1c	Glucose	C-peptide	Insulin	HOMA-IR	HbA1c	Glucose	C-peptide	Insulin	HOMA-IR
HVLT-R	r	0.012	-0.080	-0.014	-0.152	-0.160	-0.076	0.120	0.023	0.076	0.063
	p value	0.935	0.582	0.921	0.292	0.271	0.567	0.366	0.863	0.567	0.634
BVMT-R	r	-0.350	-0.238	0.000	-0.169	-0.184	-0.262	-0.002	-0.031	0.033	0.028
	p value	0.013	0.096	0.999	0.242	0.205	0.047	0.985	0.817	0.806	0.835
WMS-III SS	r	-0.295	-0.285	-0.155	-0.395	-0.370	-0.218	0.052	-0.343	-0.102	-0.072
	p value	0.037	0.045	0.283	0.005	0.009	0.097	0.695	0.008	0.442	0.590
LNS	r	-0.157	-0.092	0.055	-0.231	-0.258	-0.348	-0.134	-0.128	-0.104	-0.102
	p value	0.276	0.524	0.707	0.106	0.073	0.010	0.332	0.357	0.454	0.461
TMT-A	r	0.135	0.047	-0.050	0.091	0.081	0.330	-0.046	0.199	0.016	-0.008
	p value	0.349	0.747	0.732	0.528	0.579	0.011	0.729	0.131	0.901	0.955
BACS SC	r	-0.329	-0.250	-0.152	-0.351	-0.375	-0.176	0.048	-0.002	0.055	0.050
	p value	0.020	0.080	0.292	0.013	0.008	0.182	0.721	0.986	0.677	0.706
Fluency	r	-0.002	-0.070	0.076	-0.215	-0.202	-0.106	0.214	-0.044	0.061	0.073
	p value	0.987	0.629	0.598	0.134	0.165	0.426	0.107	0.745	0.648	0.585
NAB Mazes	r	-0.243	-0.093	0.151	0.020	0.010	-0.425	-0.067	-0.174	0.021	0.050
	p value	0.089	0.520	0.295	0.890	0.944	0.001	0.620	0.191	0.877	0.712
CPT-IP	r	-0.037	-0.042	-0.123	-0.248	-0.265	-0.262	0.049	-0.015	0.138	0.145
	p value	0.800	0.775	0.397	0.082	0.066	0.047	0.717	0.912	0.303	0.277
MSCEIT-ME	r	-0.252	-0.268	-0.038	-0.295	-0.323	-0.137	0.036	-0.155	0.024	0.018
	p value	0.078	0.060	0.793	0.037	0.024	0.318	0.797	0.259	0.863	0.897

HVLT-R, Hopkins Verbal Learning Test-Revised; BVMT-R, Brief Visuospatial Memory Test-Revised; WMS-III-SS, Corsi Block-Tapping Test; Weschler Memory Scale (3rd edition) spatial span; LNS, Letter Number Span; TMT-A, Trail Making Test part A; BACS-SC, Brief Assessment of Cognition in Schizophrenia-Symbol Coding; NAB, Neuropsychological Assessment Battery; CPT-IP, Continuous Performance Test – Identical Pairs; MSCEIT-ME, Mayer-Salovey-Caruso Emotional Intelligence Test – Managing emotions.

Pearson’s correlation analyses were used for exploring associations between HbA1c and glucose measures and cognitive tasks. Spearman’s correlation analyses were used for exploring associations between c-peptide, insulin and HOMA-IR, and cognitive tasks.

**Figure 1:**
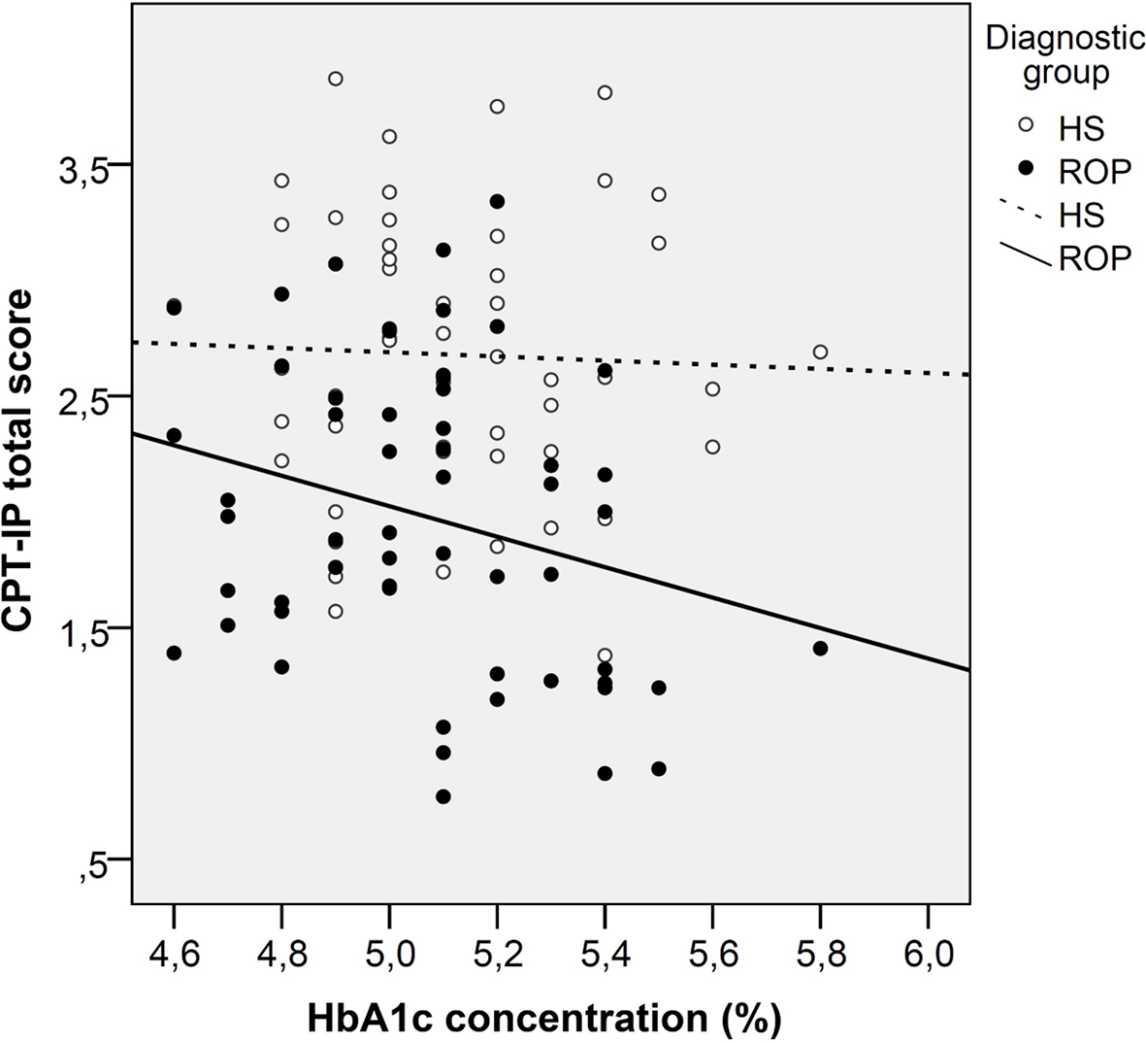
Scatterplot graph of the association between glycated haemoglobin and attention in patients with a recent-onset psychosis and healthy subjects. ROP, Recent-onset psychosis; HS, Healthy subjects.

A multivariate analysis conducted in all participants (ROP patients and HS) found a significant negative association between HbA1_c_ and cognitive functioning in five cognitive tasks dealing with executive functions, visual memory and attention/vigilance (**Table 5**). A diagnosis by HbA1_c_ interaction was found in this latter cognitive domain, which means that the pattern in the relationship between HbA1_c_ and attention/vigilance differs between ROP patients and HS: in ROP patients, higher HbA1_c_ levels are associated with impaired attention and vigilance, but this pattern does not apply to HS. This interaction is also described in **Figure 1**. As it is observed in this figure, the interaction is driven by higher HbA1_c_ values. Although there were no outliers in our sample, we repeated a sensitivity analysis excluding the two higher values (HbA1_c_= 5.8%) to explore whether the interaction was influenced for these values, and this was the case because the interaction term lost its significance when these two higher values were excluded from the analyses.

**Table 5:** Multiple linear regression analyses exploring the relationship between HbA1_c_ and cognitive abilities.

	Unadjusted model			Final model (adjusted for covariates^†^ and interactions between HbA1_c_ and diagnosis)			
	R^2^	β	p value	R^2^	β	p value	Significant interactions
HVLT-R	0.0002	0.016	0.871	0.465	-0.014	0.862	None
BVMT-R	0.038	-0.194	**0.043**	0.486	-0.189	**0.017**	None
WMS-III SS	0.041	-0.202	**0.034**	0.371	-0.143	0.100	None
LNS	0.029	-0.169	0.077	0.292	-0.170	0.065	None
TMT-A	0.02	0.143	0.137	0.474	0.160	**0.044**	None
BACS SC	0.015	-0.124	0.197	0.617	-0.136	**0.044**	None
Category fluency	0.0001	0.010	0.914	0.417	-0.014	0.864	None
NAB Mazes	0.082	-0.287	**0.002**	0.407	-0.290	**0.001**	None
CPT-IP	0.008	-0.089	0.355	0.489	0.021	0.816	ROP x HbA1_c_: β= -1.888; p=**0.016**
MSCEIT-ME	0.015	-0.121	0.208	0.337	-0.099	0.266	None

β, Standardized beta regression coefficient (for HbA1_c_); HVLT-R, Hopkins Verbal Learning Test-Revised; BVMT-R, Brief Visuospatial Memory Test-Revised; WMS-III-SS, Corsi Block-Tapping Test; Weschler Memory Scale (3rd edition) spatial span; LNS, Letter Number Span; TMT-A, Trail Making Test part A; BACS-SC, Brief Assessment of Cognition in Schizophrenia-Symbol Coding; NAB, Neuropsychological Assessment Battery; CPT-IP, Continuous Performance Test – Identical Pairs; MSCEIT-ME, Mayer-Salovey-Caruso Emotional Intelligence Test – Managing emotions.

^†^Covariates: age, sex, education level, diagnosis, antipsychotic and benzodiazepine treatment, BMI, smoking, cortisol at awakening, cortisol awakening response, cortisol levels during the day (AUC_g_).

Significant associations (p < 0.05) are represented in bold.

## Discussion

In our study that explored whether glucose metabolism parameters may contribute to the explanation, at least in part, of the cognitive deficits of individuals diagnosed with a ROP, we found that HbA1_c_ contributed to a poorer cognitive performance in domains related to processing speed, executive functions and visual memory in both the ROP patients and the HS, whereas it was associated with poorer attention and vigilance only in the ROP group.

With the main aim of a better understanding of the molecular, cellular, and other system disturbances in patients with schizophrenia, biomarkers of diagnosis, prognosis, or treatment response have been recommended (38). The study of glucose metabolism parameters in this population is of special interest since patients with schizophrenia have a three-fold risk of diabetes compared to the general population (39), and it gives us the opportunity to investigate pathogenic processes underlying both disturbances, with the aim of discovering new treatment strategies.

Biomarkers of schizophrenia have been widely used in recent years. They are frequently divided into two groups: peripheral and central biomarkers. The central nervous system and the periphery are strongly connected, a fact that has revealed the relevance of blood-based parameters as biomarkers in schizophrenia. Once again, several classifications for biomarkers have examined molecules modulating brain functions. Biomarkers have then been divided into inflammatory biomarkers, neuroendocrine biomarkers, neurotransmitters (well-documented and deeply understood), and metabolic biomarkers (38). The last may include indicators of metabolic syndrome or insulin resistance (13) that have been proven to discriminate between patients with or without metabolic syndrome. Furthermore, for many years, increased glucose concentrations, insulin resistance, and impaired glucose tolerance (13, 40) have been reported to be present in drug-naïve first-episode psychosis patients compared to HS. However, in our study, we did not find significant differences in most glucose-related parameters, except for C-peptide between ROP patients and HS.

In our study, in terms of all glucose parameters, HbA1_c_ levels were found to be associated with poorer cognitive performance, particularly in those cognitive tasks assessing executive function and visual memory in both groups, ROP patients and HS. These findings agree with a recent study carried out by Zhang et al. (24), who found this parameter to be correlated with poorer global cognition and attention in men suffering from schizophrenia. Other studies (23) have reported an association between glucose intolerance and more severe negative symptoms and poorer social cognition, although no associations were found for neurocognitive performance. There is also a meta-analysis suggesting that type 2 diabetes is associated with more severe cognitive deficits in schizophrenia (16). Interestingly, in a meta-analysis (21) exploring different glucose-related biomarkers and cognitive impairment in people with type 2 diabetes, high HbA1_c_ was negatively associated with cognitive function. Our study is in accordance with this last study, as we found a greater association for HbA1_c_ than for other fasting-related glucose parameters. These results are seemingly in agreement with a recent study investigating glucose metabolic parameters associated with cognition and white matter microstructure in healthy young populations (22). HbA1_c_ levels (even under the diagnostic values for diabetes mellitus) were inversely correlated with measures of cognitive performance. Moreover, this low-grade HbA1_c_ variation negatively affected white matter integrity, that also correlated with cognitive function. This study supports our findings related to the transdiagnostic relationship between HbA1_c_ and cognitive function, as associations between these measures were observed in both ROP patients and healthy controls.

In contrast with other cognitive domains, attention was negatively associated with HbA1_c_ in ROP patients but not in HS. It is not clear why a distinct pattern could exist between ROP patients and HS in this particular domain. Previous studies that have measured allostatic load, an index that reflects systemic biological dysregulations including glucose homeostasis parameters (glucose and insulin), have reported associations with poorer attention in ROP patients but not in HS (41). In another double-blind, placebo-controlled experimental study that assessed the effects of multiple-dose oral glucose administration on cognition in younger and older patients with schizophrenia and HS, a decrease in attentional performance at the 75 g glucose dose, when compared to placebo, was found in younger patients with schizophrenia (42). These findings suggest that glucose metabolism parameters might differentially affect ROP patients and HS in the attention and vigilance domains. However, it is also important to mention that this interaction was driven by higher HbA1_c_ values, as the interaction lost its significance when analyses were restricted to people with HbA1_c_ below 5.8%. The HbA1_c_ range of our sample was also low, which limits the possibility of finding associations between cognitive outcomes and this glucose-related biomarker. Further studies might improve this issue by including a sample with a greater proportion of patients with glucose intolerance. This can be achieved by recruiting patients with a longer duration of the illness, because pre-diabetes and diabetes might increase over time. As only 16% of patients with first episode psychosis show abnormal glucose tolerance (13), the recruitment of patients with psychotic disorders at early stages of the illness might explain the narrow range of HbA1_c_ levels in our sample.

The negative correlation between HbA1_c_ and cognitive performance found in our study may be partially explained by the fact that cognitive tasks associated with HbA1_c_ are mainly those implicating hippocampal functions and the prefrontal cortex, a hypothesis that is supported by a recent review (43). Continuous exposure to glucose and prediabetes have been significantly associated with structural brain abnormalities such as decreased brain volume and grey matter and white matter volume (44). Further, the risk of brain infarcts and decreased hippocampal volume may be associated with continuous exposure to glucose, which is reflected by higher levels of HbA1_c_. This indicator of long-term glycaemic control is also thought to impact negatively on white matter structure even in healthy individuals (22). This last study suggests that biological processes other than microvascular and macrovascular disease could be playing a role in this associations. As pointed out by Repple et al. (22), it is plausible that inflammatory processes might constitute one of several biological mechanisms potentially mediating the relationship between glycose dysregulation and brain structural damage. This is a particularly important issue to be studied because the low-grade inflammation is also found in people with psychotic disorders (45–47), even before the onset of the disease (48, 49), and is associated with poorer cognitive function (9, 50). The duration of postpandrial glucose increase, a major contributor to chronic hyperglycaemia (and higher HbA1_c_), is also thought to contribute to excessive protein glycation, generation of oxidative stress and inflammation (51). Future longitudinal studies are needed to explore whether the association between HbA1_c_ levels and impaired cognitive function could be explained by changes in inflammatory markers.

Recently, some authors have hypothesized that central insulin resistance could have an important role in the relationship between metabolic and cognitive disorders (52). There is consistent evidence pointing out that the dopamine system has an important role in glucose homeostasis control because the dopaminergic and insulin signalling pathways influence each other (53–55) and that both central nervous system insulin and striatal dopamine can regulate peripheral glucose homeostasis (56). Given the central importance of dopaminergic dysregulation, cognitive deficits, and metabolic dysfunction in schizophrenia (57–59), the potential role of central nervous system insulin signalling in the pathophysiology of schizophrenia is an interesting field to be explored. This knowledge could help in the exploration and development of future therapeutic strategies.

For most cognitive domains, with the exception of attention and vigilance, the association between HbA1_c_, and cognition was not specific for patients with ROP. Moreover, ROP patients and HS had similar HbA1_c_ levels, which were in the normal range. These findings suggest that subtle differences in HbA1_c_ could have a significant impact on cognitive processing independent of diagnosis. As our study has a cross-sectional design and includes psychotic patients who are at early stages of the disease, our study does not allow us to infer causality. Future longitudinal studies are needed to explore whether longitudinal changes in glucose metabolic parameters (mainly HbA1_c_) could contribute to cognitive impairment in patients with schizophrenia. This is an important hypothesis to be tested, as chronic antipsychotic treatment is associated with weight gain and metabolic abnormalities, including the risk of type 2 diabetes (60). If our results are replicated in longitudinal studies, therapeutic agents that improve insulin sensitivity and promote neurogenesis, such as metformin (61), could be considered cognitive enhancement options for people with ROP and higher HbA1_c_ values.

The results regarding HbA1_c_ and cognitive performance were independent of HPA axis activity, as all multivariate analyses were adjusted for cortisol levels during the day and the CAR. These two HPA axis measures have been reported to be associated with impaired cognitive functioning in ROP patients (4): a blunted CAR has been associated with poorer verbal memory in ROP patients (27), higher afternoon plasma cortisol levels are associated with poorer verbal memory (62), and a more flattened diurnal cortisol slope has been associated with poorer working memory in women with ROP (3). It is also important to control for HPA axis activity when exploring the role of glucose-related parameters on cognition because a central dysregulation of the HPA axis has been reported in type 2 diabetes and because higher morning cortisol levels are associated with poorer cognitive abilities in people with type 2 diabetes (28). In patients with type 2 diabetes, there are elevated basal plasma cortisol and ACTH levels (63–65), higher late-night cortisol (66), and increased cortisol levels following overnight dexamethasone suppression (67, 68).

Several strengths of our study should be noted. The patients in our study seemed to be representative of our catchment area, and our first-episode psychosis unit is a referral center in our area. On the other hand, to the best of our knowledge, few studies have tried to specifically investigate metabolic biomarkers for cognition in schizophrenia. Although some studies have explored the association between diabetes mellitus and poorer cognitive functioning in patients with schizophrenia (16, 24), our study is the first to specifically highlight the relationship between HbA1_c_ and cognitive function in the early stages of the psychotic illness. The search for biomarkers in cognition has been the focus of interest in research on patients with schizophrenia (50). However, in recent decades, the vast majority of studies have investigated whether neurotrophic factors or inflammatory markers may be correlated with cognitive deficits or cognitive recovery in schizophrenia (50), and metabolic parameters have been a second focus of interest.

The main limitation of our study is the cross-sectional design. Therefore, no causal relationships between glucose metabolism parameters and cognitive functioning can be inferred. The relatively small sample size limits the possibility of the conduction of specific subanalyses regarding sex differences or psychotic phenotypes. Larger studies are needed to test whether the role of HbA1_c_ on cognitive outcomes differs by subtypes of psychotic disorders. The CAR was obtained only on one day, as consensus guidelines (69) recommend obtaining repeated measures for this parameter on separate days. As in the original study (3), we administered dexamethasone to all participants, and we decided to measure the CAR only on one day. Finally, a replication dataset was not included.

In summary, the present study suggests that long-term exposure to higher glucose levels, although within the normal range, is negatively associated with cognitive performance in both ROP patients and HS in tasks dealing with executive functions and visual memory, two cognitive domains that involve the hippocampus and prefrontal cortex. Our study suggests that this association is independent of BMI and HPA axis functioning. These findings highlight the importance of the consideration of the inclusion of HbA1_c_ in future longitudinal studies exploring cognitive changes in patients with a psychotic disorder at early stages of the disease to disentangle a potential negative effect on cognitive outcomes.

## Data Availability Statement

The datasets generated for this study will not be made publicly available. The datasets are not publicly available due to privacy or ethical restrictions. However, additional analyses might be available from authors by request to the corresponding author.

## Ethics Statement

The studies involving human participants were reviewed and approved by Hospital Sant Joan Ethics Committee. The patients/participants provided their written informed consent to participate in this study.

## Author Contributions

JL, AG-Z, and EV designed the study. IM, ÁC, MS, VS-G, MA, and LO participated in the recruitment of participants. LM and EV participated in the biochemical determinations. JL and IM analyzed the data. IM and AG-R reviewed the scientific literature and wrote the first draft of the manuscript. All authors participated in the discussion of the results and approved the final manuscript.

## Funding

This work was funded in part by the Instituto de Salud Carlos III (PI10/01607; PI15/01386) and by La Fundació de la Marató de TV3 (92230). JL and IM have received an Intensification of the Research Activity Grant by the Health Department from the Generalitat de Catalunya (SLT006/17/00012 and SLT008/18/00074). JL has also received an Intensification of the Research Activity Grant by the Instituto de Salud Carlos III in 2020 (INT19/00071).

## Conflict of Interest

JL and VS-G have received honoraria for lectures or advisory boards from Jannsen, Otsuka and Lundbeck.

The remaining authors declare that the research was conducted in the absence of any commercial or financial relationships that could be construed as a potential conflict of interest.
